# Collateral damage: benchmarking off-target effects in genome editing

**DOI:** 10.1186/s13059-019-1725-0

**Published:** 2019-06-03

**Authors:** Dana Carroll

**Affiliations:** Department of Biochemistry, University of Utah School of Medicine, Salt Lake City, UT USA

## Abstract

This editorial discusses what levels of off-target effects can be tolerated in genome editing, in the context of various types of applications.

The ability to make targeted, intentional changes in chromosomal DNA using the tools of genome editing has been a tremendous boon to the practice of genetics, particularly with the advent of the easy-to-operate CRISPR platform [1]. The technology is also making its way to the clinic and the farmer’s field with applications that promise to have significant benefits for humanity.

Concerns have been raised—appropriately—that genomic sequences beyond those being targeted may also be affected in the process. As most commonly practiced, genome editing depends on a double-strand DNA break made at the intended site by a programmable nuclease—zinc-finger nuclease (ZFN), TALEN, or CRISPR-Cas [2]. If the nuclease lacks perfect specificity, secondary sites may also be cleaved, leading to unwanted mutations.

A recent article in *Genome Biology* reviewed methods for identifying secondary targets, for detecting mutations at those off-target sites, and for improving the specificity of CRISPR-Cas cleavage [3]. In general, the specificity of each of the nuclease platforms is quite good, but is good good enough?

The goal of the current exercise is to think about benchmarks for off-target effects—i.e., what levels of collateral damage can be tolerated in genome editing? There is no single answer to this question; what we care about depends on the particular application.

In the case of genetics research, where the function of a particular gene is investigated by knocking it out or introducing a variant, the effect of mutations at secondary sites can be addressed in several ways. The basic question is whether the phenotypic changes observed are due to loss of the target gene’s function alone. Independent mutations can be generated, preferably by targeting different sites in the intended target. If they all produce the same phenotype, that is supportive. Perhaps the best test is to determine whether reversing the knockout mutation or complementation with a wild-type gene reverses the phenotype completely. It is also possible with many organisms to out-cross the targeted mutation into a clean genetic background. Whole genome sequencing (WGS) can be a good option when a single individual propagates the mutation to subsequent generations. We need to keep in mind whenever WGS is used that dividing cells accumulate spontaneous mutations that are not attributable to genome editing.

Genome editing is being applied to crop plants and livestock, with the goal of establishing more secure and nutritious food sources. These applications benefit from the ability to expand favorable traits rapidly from a small number of founders via seed or semen. Individuals from the first few generations can be characterized by WGS to identify off-target mutations and by careful phenotyping to reveal any adverse effects on either the health of the organism or the quality of the food product. Incidental mutations that create such problems would not be propagated, and those that have no adverse effects can certainly be tolerated. Many existing strains of crop plants were derived by breeding selection after broad, random mutagenesis with radiation or chemicals; and those strains retain a substantial load of background mutations that are never characterized or acknowledged [4].

When it comes to clinical applications of genome editing, we need to make some distinctions. Each somatic therapy for a specific disease will likely have its own particular set of off-target concerns, so it will have to be determined in each case what the risks are. Take the developing genome editing therapies for sickle cell disease, for example. Both the approach that intends to correct the specific sickle mutation [5] and the one that works by reactivating the expression of fetal beta-globin [6] propose to do the editing in hematopoietic stem cells (HSCs) ex vivo and to restore them to the patient from which they came. In this situation, we care most about what could go wrong in those cells and in the lineages to which they contribute. It makes perfect sense to identify all the candidate secondary targets and to assess what their impact might be. Mutations in a tumor suppressor gene could lead to leukemia and should be strictly avoided. Mutations in a muscle-specific gene, like dystrophin, which is not expressed or required in the hematopoietic lineages (as far as we know), are probably tolerable. Conversely, mutation of a beta-globin gene seems minimally threatening in a somatic therapy for muscular dystrophy. In a few words, the hazardous target comprises only a subset of the whole genome.

Probably the best way to proceed in the sickle cell therapies is to identify potential targets with Digenome-seq, GUIDE-seq, or DISCOVER-seq [3, 7], and to determine the level of mutagenesis at those sites by targeted PCR and deep sequencing. In addition, oncogene and tumor suppressor gene collections can be examined with targeted sequencing. WGS is not a great choice because adequate sequencing depth is difficult to reach. Tumor-inducing mutations would give those cells a growth advantage in the patient, so they would not have to be present at high frequency in the treated cells to emerge later. This is exactly what happened in some SCID-X1 and Wiscott–Aldrich patients who developed leukemia after receiving a therapeutic replacement gene [8]. In those cases, the viral vector integrated near an oncogene and induced its untimely expression. The proportion of HSCs that had such an integration event was estimated at 10^− 4^ or less, well below the detection limit of WGS. Activation of the growth-promoting gene, however, provided a significant advantage, and those cells began to take over after an extended period.

As illustrated in the sickle cell example, genome editing is well suited to collaborate with stem cell approaches to therapy. No other tissues contain natural stem cells as well-characterized or as easily manipulated as HSCs. As an alternative, researchers are exploring the prospect of using embryonic stem cells (ESCs) or induced pluripotent stem cells (iPSCs) in attempts to repopulate diseased or damaged tissues. This has the advantage that the stem cells can be prepared from each patient to minimize the likelihood of rejection. At present, however, the ability to control or predict the differentiation fate of such cells once returned to the body lags behind the ability to alter their genomes.

With in vivo somatic therapies, such as that being pursued for Hunter syndrome [9], the risks of off-target mutagenesis are amplified compared to ex vivo methods. Whether the nuclease is delivered in a viral vector or nanoparticle, its activity will not be restricted to a single cell type or tissue, and the milieu of the receiving cells will differ substantially from any provided in prior in vitro tests. Still, identifying the candidate targets and evaluating them thoroughly should provide substantial confidence for proceeding in cases where alternative treatments are lacking. A positive aspect of all somatic therapies is that only the treated individual is at risk for adverse effects, which may be reversible, and the therapy can be stopped before other patients are treated.

Finally, prospects for human germline genome editing have been brought into sharp focus by the report last November of the first “CRISPR babies” [10]. In this case, the hazardous target does comprise the whole genome, because all cells and tissues in the body will be affected during the development and life of the treated individual. Not only coding sequences, but non-coding sequences of unknown, possibly regulatory function are at risk. The off-target analysis will have to be comprehensive, and we will still be left with uncertainties.

Without addressing the ethical aspects of reproductive editing, there is some positive news on the technical side. If the editing is done at the one-cell stage, only two genomes are at risk. This is unlike an ex vivo HSC therapy, where infusion of 10^7^ treated cells would reflect risk to 2 × 10^7^ genomes. If, for example, a CRISPR-based treatment had an efficiency of 90% at the intended target and even as high as a 5% rate at a single secondary target, most of the treated embryos would have only the desired genome alteration. Even if several sites were identified, but with realistically lower frequencies, the situation would be favorable. In addition, as long as all editing occurred at the one-cell stage, evaluation of a few trophoblast cells at the blastocyst stage prior to implantation would reveal the status of all cells in the developing embryo, and mis-edited ones could be discarded. The risks, however, are huge and largely unpredictable, so the benefits would have to be undeniable. In addition, of course, all on- and off-target modifications, whether beneficial or detrimental, will be passed on to subsequent generations, which places an even higher standard for safety and efficacy on the process.

## Concluding remarks

In every application of genome editing, a thorough risk:benefit analysis should be applied. Both sides of that comparison will be particular to the situation at hand and, therefore, difficult to generalize. With the tools currently available for analysis and avoidance of unwanted genomic cleavage, most applications will not be limited by off-target mutagenesis, as long as users are diligent in their approach.

## Abbreviations

HSC: Hematopoietic stem cell
WGS: Whole genome sequencing
